# Mucoadhesive Nanoemulsion Enhances the Antineoplastic Potential of Naringenin in Bladder Cancer Cell Lines

**DOI:** 10.1021/acsomega.5c11886

**Published:** 2026-05-19

**Authors:** Kamila de Fátima da Anunciação, Tatiane Roquete Amparo, Lucas Resende Dutra Sousa, Viviane Flores Xavier, Paula Melo de Abreu Vieira, Orlando David Henrique dos Santos, Geraldo Célio Brandão, Glenda Nicioli da Silva

**Affiliations:** † Postgraduate Program on Biological Sciences, Institute of Biological Sciences, Federal University of Ouro Preto, 35400-000 Ouro Preto, Brazil; ‡ Postgraduate Program on Pharmaceutical Sciences, Federal University of Ouro Preto, 35400-000 Ouro Preto, Brazil

## Abstract

Naringenin exhibits anticancer properties against various types of cancer. Bladder cancer is among the most prevalent cancers worldwide and is characterized by a high recurrence rate because of resistance to standard chemotherapy. Nanotechnology represents a prospective approach to counteracting the challenges associated with the therapeutic application of naringenin in the treatment of bladder cancer. The aim of this study was to evaluate the antineoplastic activity of naringenin in high-grade bladder cancer cell lines (J82 and UM-UC-3) and to develop nanoemulsions. The assessment of cellular toxicity was conducted using the sulforhodamine B method, clonogenic survival using crystal violet staining, cell migration by the wound healing assay, changes in the cell cycle using flow cytometry, and expression of the specific long noncoding RNAs *RP11-363E7.4* and *SBF-AS1* by quantitative reverse transcription polymerase chain reaction (RT-qPCR). Nanoemulsions with and without chitosan were developed using the phase inversion method and characterized by measuring particle size as well as release profile, encapsulation efficiency, and mucoadhesion in vitro and ex vivo. Naringenin significantly inhibited cell proliferation, viability, clonogenic survival, and migration and altered the cell cycle in both bladder cancer cell lines. Moreover, naringenin increased the expression of the long noncoding RNA *RP11-363E7.4* in the UM-UC-3 cell line. The nanoemulsions were found to be nanometric, monodisperse, stable, sustained-release systems, and exhibited adequate encapsulation efficiency. In addition, the formulations increased the cytotoxicity of naringenin, and the chitosan-containing nanoemulsion demonstrated mucoadhesion without ex vivo toxicity. Consequently, naringenin and nanoemulsions may represent promising alternatives for the development of therapeutic agents for the treatment of bladder cancer.

## Graphical abstract

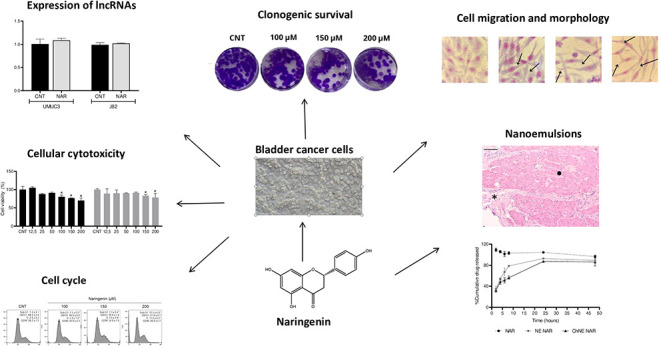

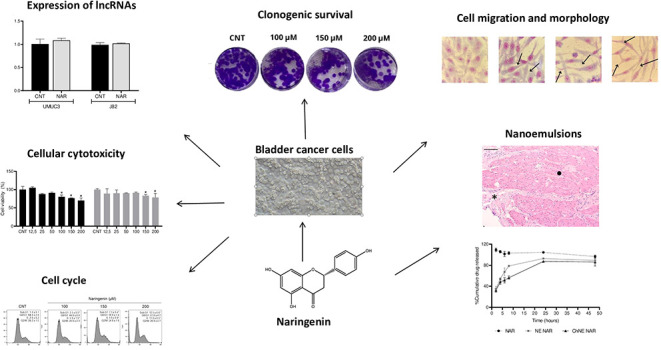

## 1 Introduction

A diet rich in fruits and vegetables can reduce the risk of some types of cancer, and this effect is attributed, in part, to natural polyphenols.^1^ Natural polyphenols are a group of secondary plant metabolites that range from small molecules to highly polymerized compounds and are widely present in foods and drinks of plant origin, such as fruits, vegetables, spices, soybeans, and nuts.^1^ Naringenin, a polyphenol belonging to the flavonoid class, found predominantly in grapefruit, lemons, oranges, and tomatoes, has attracted attention for its protective effects, which include cardioprotective, antioxidant, hepatoprotective, antidiabetic, nephroprotective, antimicrobial, neuroprotective, and anticancer properties.^2^ Research has shown that naringenin can inhibit the proliferation and migration of various types of cancer, including breast, lung, stomach, leukemia, lymphoma, and skin cancers.^2^ Therefore, it may offer a potential therapeutic strategy for antineoplastic treatment.^2^

Cancer is one of the leading causes of death, responsible for almost 10 million fatalities in 2020, as reported by the World Health Organization.^3^ Bladder cancer is the most common malignant neoplasm of the urinary tract and is among the most prevalent cancers worldwide.^4^ It is categorized into nonmuscle-invasive bladder cancer (NMIBC), which includes low-grade tumors, and muscle-invasive bladder cancer (MIBC), which encompasses high-grade tumors.^5^ In 2020, approximately 573 278 people were diagnosed with bladder cancer, and this number is expected to double by 2040, according to the World Health Organization.^5^ The main risk factor for bladder cancer is smoking, with the main symptoms including blood in the urine, dysuria, and increased urinary frequency.^6^ The disease is more prevalent in men, especially among those over 55 years of age.^5^

Treatment for bladder cancer usually involves surgery, chemotherapy, and radiotherapy. For nonmuscle-invasive tumors, immunotherapy with BCG has become the standard treatment. On the other hand, invasive tumors are usually treated with neoadjuvant therapy using cisplatin, often in combination with other agents such as gemcitabine (GC) or the MVAC regimen, which includes methotrexate, vinblastine, doxorubicin, and cisplatin, followed by radical cystectomy.^7^ However, treatment challenges persist, including resistance to chemotherapy, nonresponsiveness, a high recurrence rate, and drug toxicity, which can negatively affect patients’ quality of life and survival.^8^ These problems have stimulated research into new therapeutic options, with natural products such as flavonoids being widely studied. These compounds have various physiological activities, including the inhibition of cell growth.^8^

Long noncoding RNAs are frequently found deregulated and are linked to carcinogenesis, aggressiveness, and chemoresistance in a variety of tumors. LncRNAs SBF2-AS1 and RP11-363E7.4 are implicated in carcinogenesis. Long noncoding RNAs (lncRNAs) are involved in gene regulation processes, and many of them show abnormal expression in cancer and play essential roles in its progression, occurring both as oncogenes and as tumor suppressors.^9^ Some lncRNAs, such as SBF2-AS1 and RP11-363E7.4, are implicated in the carcinogenesis of bladder tumors.^10^

Because of its hydrophobic nature, naringenin has some limitations regarding its therapeutic use. These limitations include low aqueous solubility, low oral bioavailability, and instability.^2^ These issues can be addressed by developing nanocarriers such as nanoemulsions. Nanoemulsions o/w are prepared by combining oils and surfactants, which have the unique ability to form fine colloidal dispersions of oil in water.^2^

In addition to these components, polymers can be added to nanoemulsion formulations to enhance their properties. Chitosan, a natural polymer, is widely used in nanocarrier development due to its biodegradable, biocompatible, hydrophilic, nontoxic, and mucoadhesive properties.^11^ Therefore, chitosan-based nanoemulsions may be an effective way to deliver naringenin for the treatment of bladder cancer via an intravesical route.

A study involving TSGH-8301 bladder cancer cells indicated that naringenin reduced cell viability and migration.^12^ Other studies have demonstrated that chitosan nanoparticles increase the antitumor efficacy of naringenin in lung and breast cancer.^11,13^ However, the impact of naringenin on lncRNA expression and the effectiveness of mucoadhesive nanoemulsion in treating bladder cancer have yet to be elucidated. Therefore, this study aimed to evaluate the antineoplastic activity of naringenin on high-grade bladder cancer cells in vitro, as well as the development of nanoemulsions.

## 2 Results

### 2.1 Cell Cytotoxicity

Naringenin reduced cell viability in the two cell lines studied. This effect was observed to predominate at higher concentrations at both times, independent of the methodology used (Figure 1). The comparison of the methods revealed that the sulforhodamine B (SRB) assay exhibited high levels of cellular toxicity at the higher concentration, with a more dose-dependent effect observed in comparison to the XTT assay. The concentrations of 100, 150, and 200 μM were chosen for the next assays.

**Figure 1:**
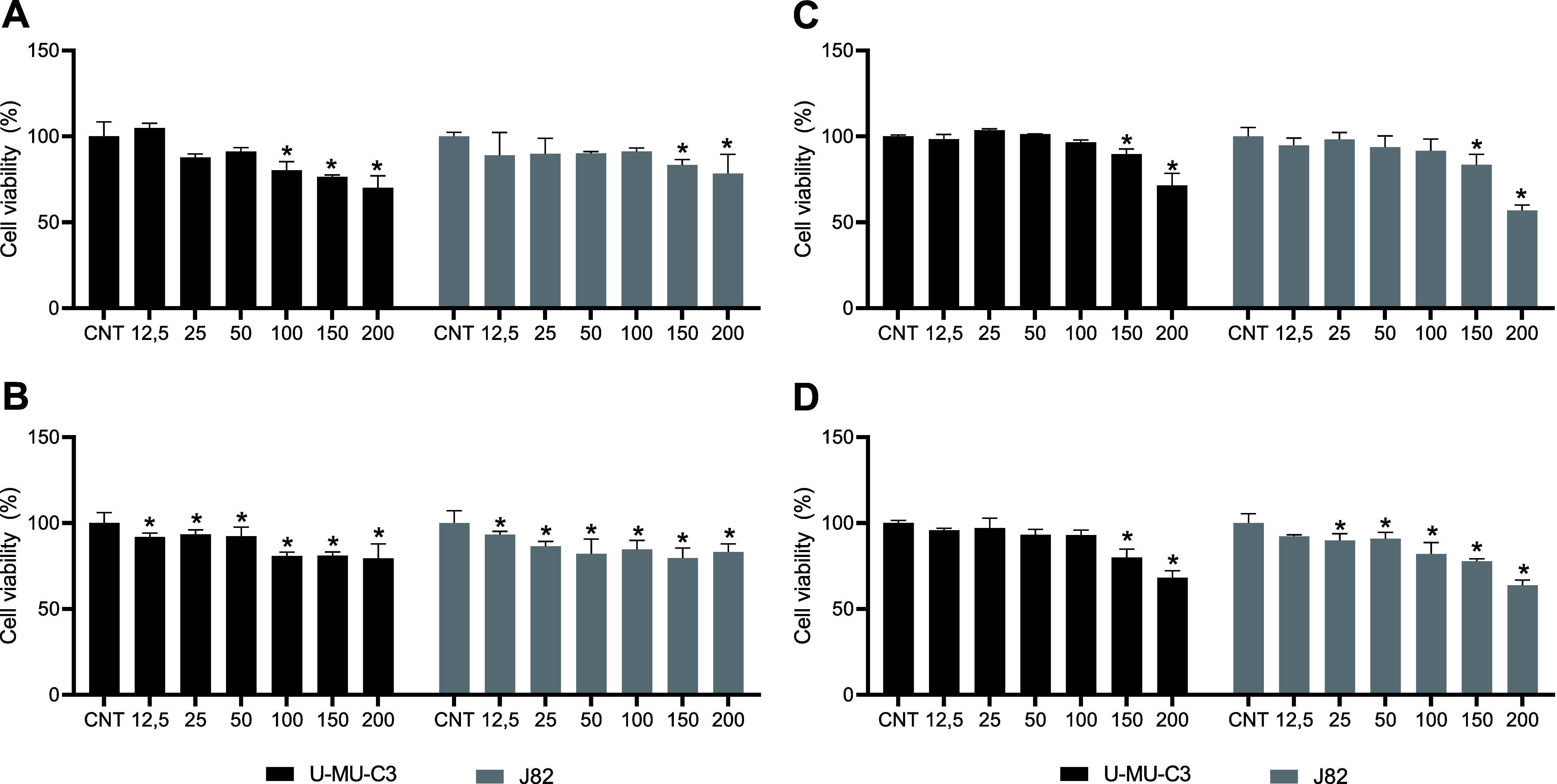
Viability of U-MU-C3 and J82 cell lines after 24 h (A, C) and 48 h (B, D) of treatment by XTT (A, B) and sulforhodamine B (C, D) assays. (*) indicates a significant difference compared to untreated cells (CNT) (*p* ≤ 0.05), according to one-way analysis of variance (ANOVA).

### 2.2 Clonogenic Survival

The clonogenic survival assay showed a reduction in colony formation after treatment with naringenin at the three concentrations chosen for both cell lines, as shown in Figure 2A,B.

**Figure 2:**
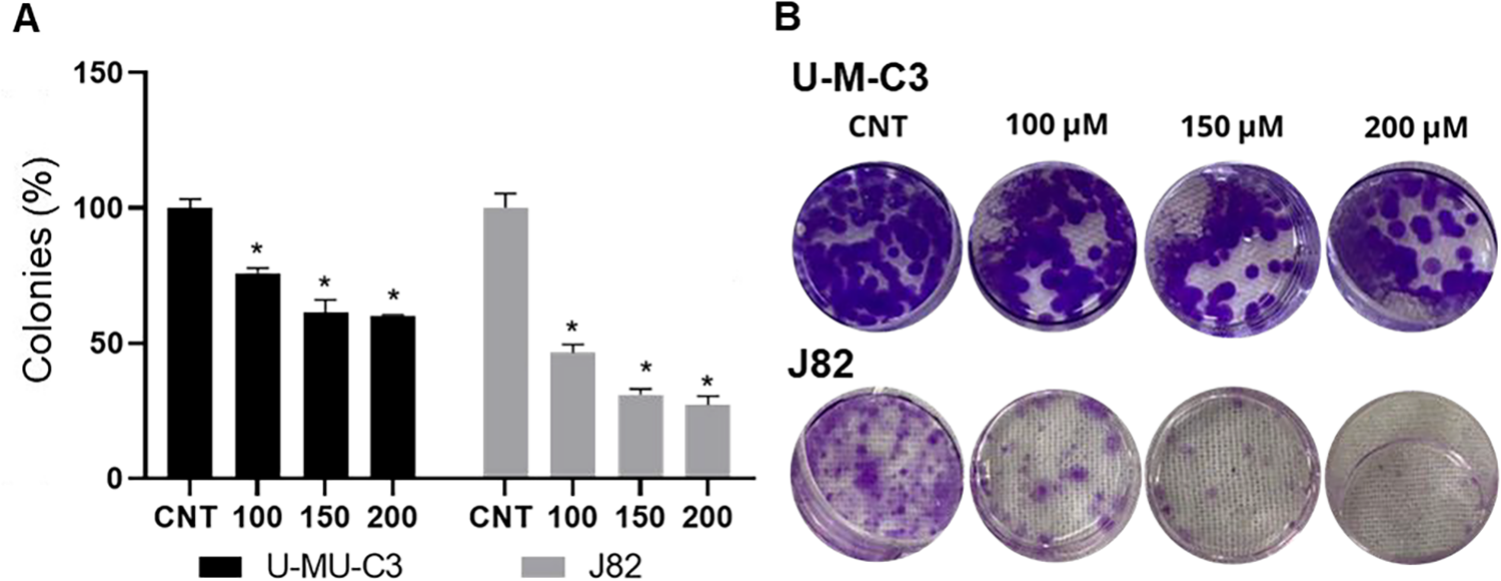
Percentage (A) and images (B) of colony formation of the U-MU-C3 and J82 cell lines after 48 h of treatment with 100, 150, and 200 μM naringenin. (*) indicates a significant difference in relation to untreated cells (CNT) (*p* ≤ 0.05), according to one-way ANOVA.

### 2.3 Cell Migration and Morphology

Naringenin inhibited migration in U-MU-C3 cells at concentrations of 150 and 200 μM and in J82 cells at all three concentrations at 24 h. At 48 h, cell migration was inhibited by the concentration of 200 μM in U-MU-C3 cells and by the concentrations of 150 and 200 μM in J82 cells. In terms of cell morphology, cell death and debris were observed at all concentrations (Figure 3).

**Figure 3:**
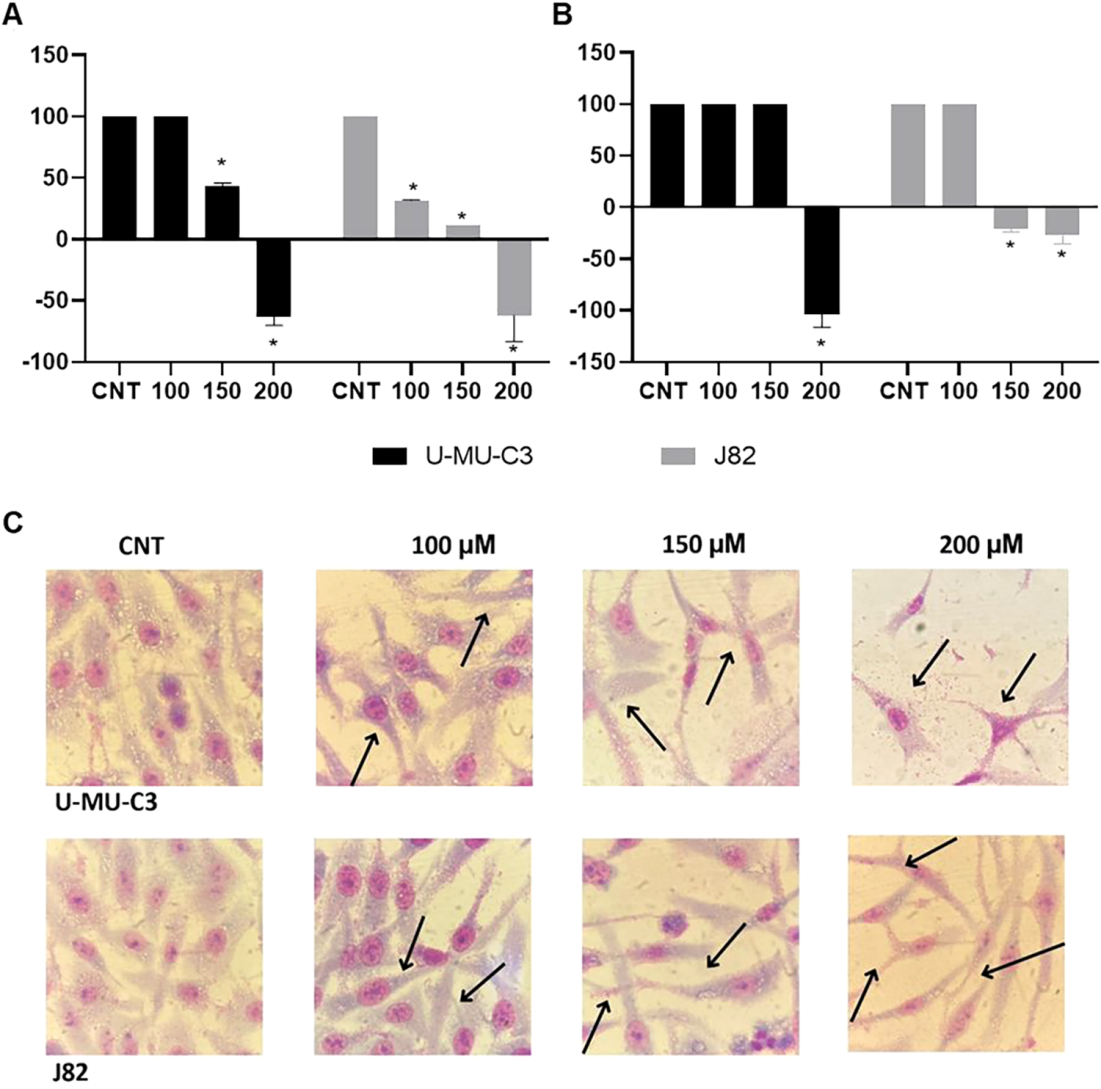
Cell migration after 24 h (A) and 48 h (B) of treatment and cell morphology (C) after 48 h of treatment with naringenin of the U-MU-C3 and J82 cell lines. Black arrows indicate elongated cells and cell debris (C). (*) indicates a significant difference compared to untreated cells (CNT) (*p* ≤ 0.05), according to one-way ANOVA.

### 2.4 Cell Cycle

After 48 h of treatment with naringenin, U-MU-C3 cells showed an increase in sub-G1 and S phase content at concentrations of 150 and 200 μM, along with a decrease in G0/G1 phase content at the same concentrations and a reduction in G2/M phase content at 200 μM. In J82 cells, an increase in sub-G1 and S phase content and a decrease in G0/G1 phase content were observed across all three concentrations tested (Figure 4).

**Figure 4:**
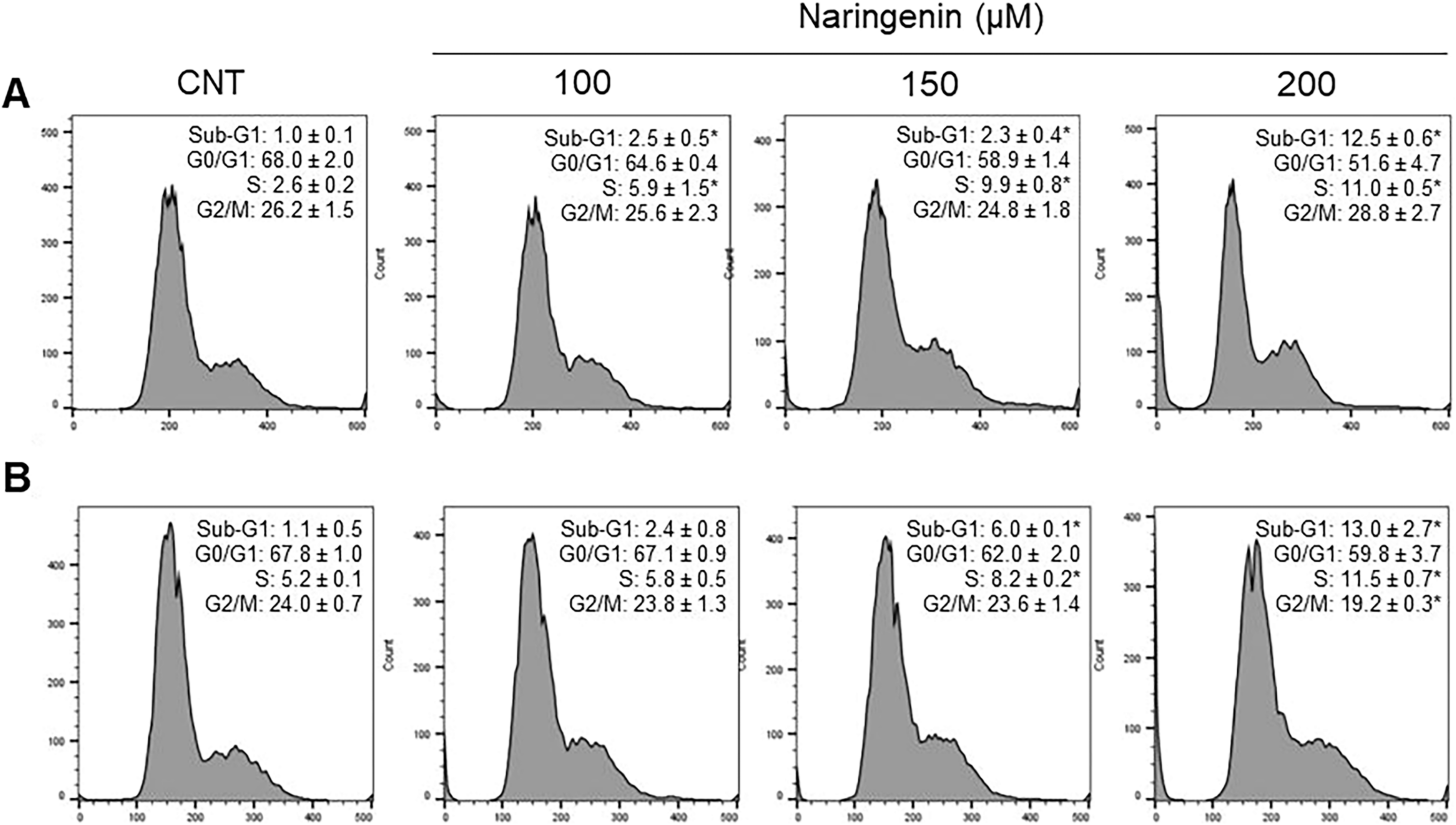
Histograms of the cell cycle analysis of the J82 (A) and U-MU-C3 (B) cells after 48 h of treatment with naringenin. (*) indicates a significant difference compared to untreated cells (CNT) (*p* ≤ 0.05), according to the analysis carried out by one-way ANOVA.

### 2.5 lncRNA Expression

Increased expression of the long noncoding RNA RP11-36E7.4 was observed in U-MU-C3 cells. J82 cells showed no difference in the expression of the long noncoding RNAs studied (Figure 5).

**Figure 5:**
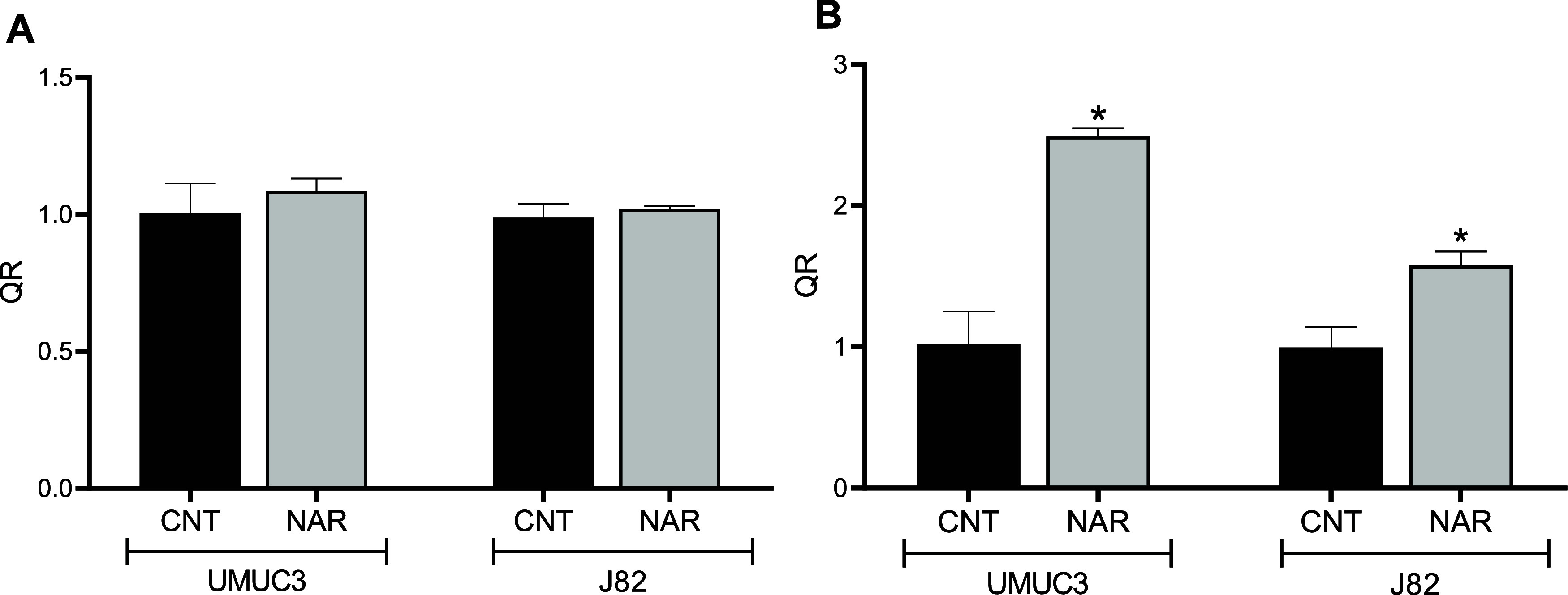
Relative expression of the lncRNAs *SBF2-AS1* (A) and *RP11-36E7.4* (B) in the U-MU-C3 and J82 cells after treatment with 200 μM naringenin (NAR) for 48 h. (*) indicates a significant difference compared to untreated cells (CNT) (*p* ≤ 0.05), according to the *t* test.

### 2.6 Characterization of the Nanoemulsions

The developed formulations exhibited a mean particle size of less than 100 nm and a polydispersity index (PDI) value of less than 0.3 (Table 1). These parameters remained unchanged during the 60-day stability evaluation period (Figure 6).

**Figure 6:**
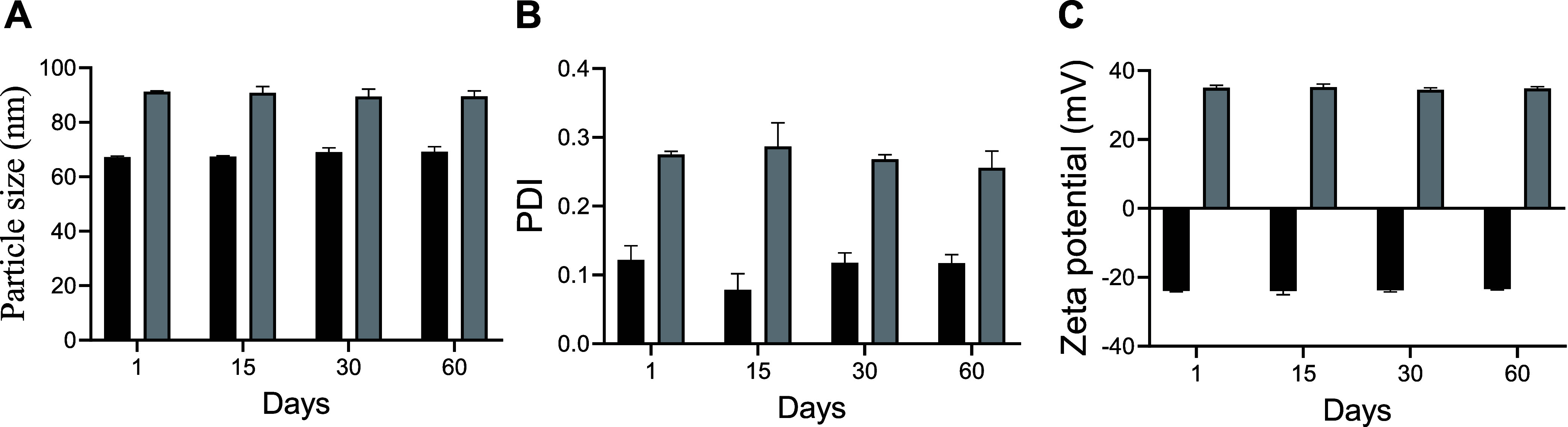
Evaluation of formulation stability by measuring the average particle size (A), polydispersity index (PDI) (B), and ζ potential (C) at 1, 15, 30, and 60 days. NE NAR: nanoemulsion comprising naringenin; ChNE NAR: nanoemulsion consisting of naringenin and chitosan.

**Table 1:** Particle Size, Polydispersity Index (PDI), and ζ Potential of the Formulations Developed with Naringenin^a^

formulation	size (nm)	PDI	ζ potential
NE NAR	67.24 ± 0.42	0.122 ± 0.034	–23.9 ± 0.3
ChNE NAR	91.33 ± 0.24	0.275 ± 0.021	35.0 ± 1.2

a NE NAR-nanoemulsion; ChNE NAR-nanoemulsion with chitosan.

The method used to evaluate the encapsulation efficiency (EE) and release profile was considered selective, precise, accurate, and linear, as shown in Table 2.

**Table 2:** Semi-Validation Parameters for Naringenin Quantification

Selectivity	
purity angle	0.241
purity threshold	0.545

Linearity	
*r* ^2^	0.9946
equation	*y* = 69,924*x* – 135,025
significance	*p* = <0.0001 (*a* ≠ 0)
linearity	*p* = 1.0000 (linear)

Precision (Repeatability)	
concentration (μg/mL)	RSD
2	1.73
8	4.65
20	0.80
100	0.41
50	1.24

Accuracy	
concentration (μg/mL)	RSD
2	1.73
10	0.41
50	1.23

With respect to the encapsulation efficiency, the values obtained for NE NAR and ChNE NAR were 99.11 and 98.49%, respectively.

The release of the nanoemulsions and free naringenin is shown in Figure 7. Free naringenin solubilized in dimethyl sulfoxide (DMSO) reached maximum release (100%) in 2 h, while NE NAR and ChNE NAR achieved maximum release (92.96 and 87.40%, respectively) in 24 h. The maximum release was sustained until the conclusion of the study period, which was 48 h for both free naringenin and the nanoemulsions.

**Figure 7:**
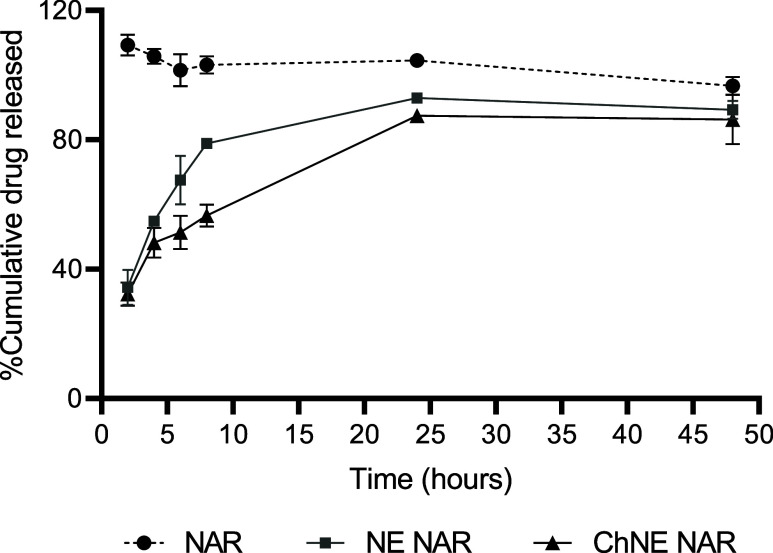
In vitro release of nanoemulsions and free naringenin. NE NAR: nanoemulsion comprising naringenin; ChNE NAR: nanoemulsion consisting of naringenin and chitosan.

### 2.7 Cytotoxicity and Mucoadhesion of the Nanoemulsions

The study found that both developed nanoemulsions exhibited increased cytotoxicity compared to free naringenin, with the nanoemulsion containing chitosan demonstrating the greatest increase (Table 3).

**Table 3:** Cytotoxicity of Naringenin and Its Nanoemulsion (NE NAR) and Nanoemulsion with Chitosan (ChNE NAR)^a^

	CC_50_ (μM)	
	U-MU-C3	J82
NAR	167.0 ± 5.4^a^	127.6 ± 8.9^a^
NE NAR	73.1 ± 3.2^b^	72.0 ± 2.7^b^
CH NE NAR	56.6 ± 3.1^c^	47.3 ± 3.9^c^

a CC_50_: Cytotoxic concentration for 50% of cells expressed as equivalent to naringenin concentration. Same lowercase letters indicate no significant statistical difference in the same column (*p* < 0.05) by one-way ANOVA test followed by a Tukey’s post-test.

The nanoemulsion containing naringenin exhibited mucoadhesion, with a substantial alteration in ζ-potential following incubation with mucin in the in vitro test (Figure 8A) and enhanced retention after washing the mucosa with artificial urine in the ex vivo test (Figure 8B,C), in comparison to the nanoemulsion without chitosan.

**Figure 8:**
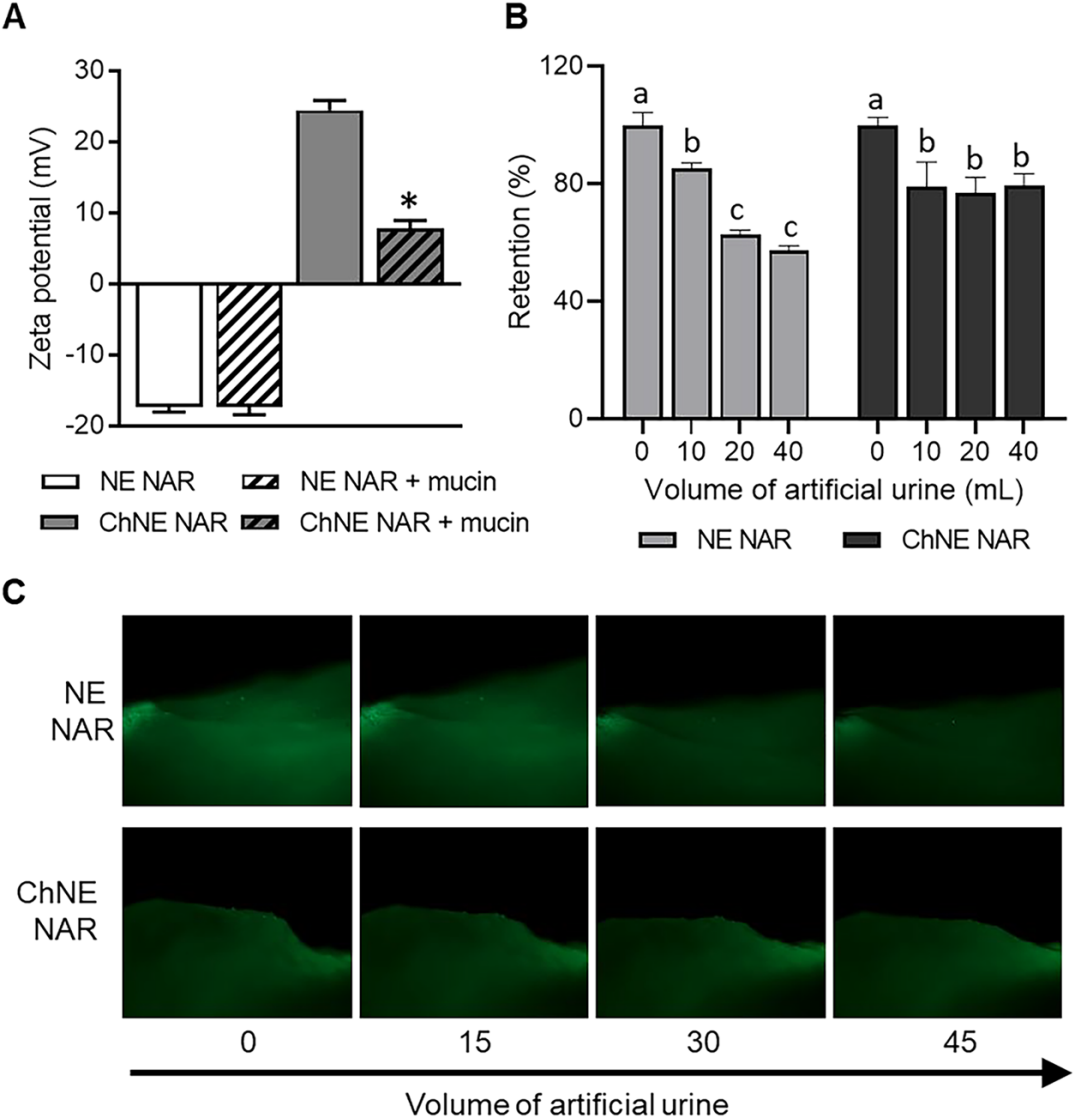
Mucoadhesion analyzed by in vitro (A) and ex vivo (B, C) assays of naringenin-containing nanoemulsions with or without chitosan (NE NAR and ChNE NAR, respectively).

Qualitative histopathological analyses were also performed in the ex vivo test, revealing no observable morphological alterations in bladder tissues treated with either formulation when compared with the control group (Figure 9). The lamina propria, comprising loose connective tissue, and the muscular layer exhibited the preservation of their characteristics following treatment with the formulations.

**Figure 9:**
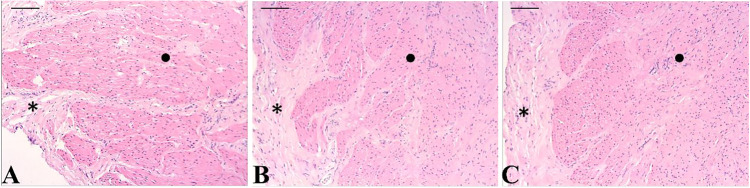
Photomicrographs of histological sections of the pig bladder (*Sus scrofa* *domesticus*) treated with the formulations. (A): Control; (B) NE NRG; and (C) ChNE NRG. Hematoxylin–eosin (HE). Bar = 50 μm. * Lamina propria and ^•^ muscular layer.

## 3 Discussion

Naringenin is a natural compound with potential activity in cancer treatment.^2^ This flavonoid has been shown to have inhibitory effects on the proliferation and migration of various types of cancer, including bladder cancer.^2^

In this study, it was observed that naringenin had inhibitory effects on cell viability and migration in the cell lines studied, J82 and U-MU-C3. Cell cytotoxicity assesses the number of living cells in a given population, and measuring the number of proliferating cells is used as a vital indicator of cell survival or death in response to drugs or chemical agents.^14^ The differences observed between the results obtained by using the two methods can be attributed to the interaction of naringenin with the XTT reagent. This phenomenon has previously been reported for other phenolic compounds, which are capable of directly reducing tetrazolium salts such as XTT and MTT, leading to unreliable data.^15,16^ Therefore, alternative methodologies, such as the sulforhodamine B (SRB) assay, which do not rely on redox reactions, are considered more reliable for evaluating the cytotoxicity of compounds such as flavonoids.^16^

A study by Liao et al. with TSH-8301 bladder cancer cells also demonstrated this same inhibitory effect on cancer cells after treatment with naringenin, suppressing the migration of bladder cancer cells in a dose-dependent manner. The TSH-8301 cells contain endogenous wild-type TP53 genes.^17^ U-MU-C3 and J82 are high-grade bladder tumor cells that have mutations in the TP53 gene.^18^ The TP53 gene is considered the most important tumor suppressor gene.^19^ Mutations in TP53 are found in approximately 50% of tumors, and these mutations are under positive selection, as they are highly beneficial for cell survival and proliferation.^19^ This highlights the importance of studying cancer cell lines with mutations in the TP53 gene and shows that naringenin has antiproliferative effects independent of the TP53 status. Moreover, changes in cell migration observed after treatment show that naringenin may be important for the metastasis process. The movement of cancer cells to peripheral organs and their resulting destruction constitute a primary cause of cancer-related morbidity and mortality.^20^

A similar reduction in colony formation was observed in a study of breast cancer cells treated with naringenin, as reported by Abaza et al.^21^ The clonogenic cell survival assay or colony formation assay is based on a cell’s ability to proliferate into a large colony or clone. Clonogenic survival is assessed based on a relationship between the dose of the agent used and the proportion of cells that maintain their ability to reproduce.^14^ The clonogenic survival result confirms the cell viability results found through the cytotoxicity test, however, indicated a more pronounced and dose-dependent effect. The clonogenic survival assay is regarded as the most suitable for evaluating activity in tumor cell lines, as it also determines whether the investigated active ingredient leads to cell damage that impedes proliferation.^22–24^ Furthermore, cells that are unable to generate colonies under optimal conditions are also less likely to develop tumors.^22^

The inhibitory effects of naringenin on viability, cell migration, and proliferation may be associated with the regulation of signaling pathways, including the AKT pathway, activity of nuclear transcription factors, and metalloproteinase-2 effects already reported by studies with other cancer cells.^12^

With regard to cell morphology, the morphological changes found in the cells and the low cell density after treatment with naringenin are suggestive of cell cycle arrest.^25^ In fact, the inhibitory effects of naringenin were also observed in the cell cycle assay. It was observed that, for both cells, there was an increase in sub-G1 content, which indicates cell death.^26^ Naringenin-induced apoptotic cell death has been demonstrated in several studies, including those on bladder and breast cancer cells. An in vitro study by Liao et al.^12^ with TSH-8301 bladder cells demonstrated that naringenin exerts an inhibitory effect on the Akt signaling pathway and on the nuclear factor NF-κB, which play a pivotal role in cell proliferation and migration, as well as in apoptosis. The findings from an experimental study by Rajamani et al.^13^ demonstrated the antitumor effects of naringenin nanoparticles in MCF-7 breast cancer cells, characterized by elevated ROS levels, glutathione (GSH) attenuation, and caspase-3 activation, culminating in the induction of apoptosis. Despite the extant literature on the subject, experiments with the cell lines included in this study should be performed in order to identify the mechanisms by which naringenin induces cell death in bladder cancer cells.

In addition, there was cell cycle arrest in GO/G1 and G2M, results also found by Arul and Subramanian^27^ who showed that naringenin is able to inhibit the proliferation of liver cancer cells. In a study carried out with triple-negative breast cancer cells, cell cycle arrest was observed in the G0/G1 phase, and apoptosis was found in all treatments of different concentrations of naringenin, with a reduced quantity of viable cells.^28^

Long noncoding RNAs (lncRNAs) are involved in gene regulation processes, and many of them show abnormal expression in cancer and play essential roles in its progression, occurring both as oncogenes and tumor suppressors.^9^ Some lncRNAs, such as SBF2-AS1 and RP11-363E7.4, are implicated in the carcinogenesis of bladder tumors.^10^ The SBF2-AS1 lncRNA has been associated with cell proliferation and a worse prognosis in various types of tumors, including bladder cancer, as observed by dos Anjos Oliveira et al.^29^ In this study, it was observed that after treatment with naringenin, there was no change in the expression of this lncRNA. However, treatment with naringenin resulted in a notable elevation in RP11-363E7.4 expression levels in the U-MU-C3 cell line. An increase was also observed in J82 cells, although it was not statistically significant. Chen et al.^30^ reported that the lncRNA RP11-363E7.4 may function as a tumor suppressor, being associated with reduced proliferation, migration, and invasion, as well as increased cell death. In this context, the increased expression of RP11-363E7.4 observed in our study may be associated with the antiproliferative effects of naringenin in bladder cancer cells, although further functional studies are required to establish a causal relationship.

Notwithstanding the findings presented in this study and in the referenced literature regarding the effects of naringenin, the clinical utilization of this flavonoid is constrained by its inherent characteristics, including low aqueous solubility, limited oral bioavailability, and instability. These properties impede its effective medical application.^2^ However, these barriers can be overcome with formulations, such as nanoemulsions.

In this study, two types of nanoemulsions were developed: one containing chitosan (NE NAR) and another devoid of chitosan (ChNE NAR). These formulations were developed to maximize the amount of encapsulated drug while respecting solubility and stability limits, with the aim of permitting greater dosage flexibility. This approach is particularly important, given the absence of clinical studies of naringenin administered intravesically to establish the doses. The execution of preclinical in vivo studies and clinical studies will enable the determination of the effective dosage.^31^ Subsequent adjustments to the formulation can be made with consideration for the dose and volume to be administered. With regard to the excipients utilized, DMSO was incorporated with the objective of enhancing the solubility of naringenin and facilitating its penetration into the bladder epithelium. DMSO is utilized in an intravesical treatment, facilitating drug penetration. Research conducted by Chen et al.^32^ and Sogutdelen and Citamak^33^ demonstrates the absence of toxicity and increased penetration with 50% DMSO in vivo in humans and dogs. However, lower doses (10% DMSO) have been shown to increase permeation,^34^ and this concentration was selected for the nanoemulsions. However, this concentration of DMSO did not affect the results obtained in this study because the formulation was diluted in the culture medium, and the final concentration of this solvent was below 1%.

According to Seibert et al.,^35^ both developed formulations can be classified as nanometric with a homogeneous particle size distribution due to their size being smaller than 100 nm and a PDI lower than 0.3. The small particle size may contribute to increased nanoemulsion permeation due to the large surface area provided.^36^ The increased availability of the drug is attributed to enhanced contact with the urothelial surface, a consequence of its reduced size.^36^

The results of the stability study of naringenin-containing nanoemulsions demonstrated stability throughout the stipulated time period for this analysis. In addition to stability, the nanoemulsions exhibited high encapsulation efficiency and prolonged release compared with free naringenin. Nanoemulsions are classified as sustained drug delivery systems wherein the oil and emulsifier layers function as release barriers. Sustained intravesical administration has been demonstrated to ensure the continuous presence of drugs in the bladder. Consequently, the intravesical delivery of drugs in nanoemulsion systems can enhance treatment efficacy by prolonging the duration of direct contact between the drug and the urothelium.^36^

In addition to prolonged release, mucoadhesion also favors intravesical treatment. In vitro and ex vivo tests demonstrated that the ChNE NAR system is mucoadhesive, thereby augmenting the drug’s residence time in the urothelium and impeding its elimination through urination.^37^ Chitosan’s mucoadhesive potential stems from electrostatic interactions between its positively charged amino groups and negative sialic acid residues in mucus.^38^

Furthermore, the encapsulation process through nanoemulsion technology has been shown to enhance the antiproliferative activity of naringenin, thereby amplifying its cytotoxic effect. This heightened activity may be attributable to enhanced drug solubility and a small particle size. A comparative analysis of the two nanoemulsions revealed that the formulation comprising chitosan (ChNE NAR) exhibited a superior activity. Formulations containing chitosan have been demonstrated to facilitate drug entry into cells due to electrostatic interactions between its positively charged surface and the negatively charged cell membrane.^39,40^ With regard to the safety of these nanoemulsions, the ex vivo findings demonstrated a favorable safety profile for intravesical use, as evidenced by the absence of tissue damage.^40^

## 4 Conclusion

Naringenin has an antitumor effect against bladder cancer cells by inhibiting cell proliferation and migration and inducing cell cycle changes. Its mechanism of action includes the modulation of lncRNA RP11-363E7.4 expression. The antiproliferative effects were enhanced by encapsulating the compound in nanoemulsions. Notably, the nanoemulsion composed of chitosan exhibited superior cytotoxicity and mucoadhesion. Consequently, this flavonoid and its chitosan nanoemulsions can be regarded as promising therapeutic strategies for the intravesical treatment of bladder cancer. It is imperative to emphasize that further studies are required to substantiate the proposed mechanism of action for this flavonoid, in addition to conducting in vivo studies to assess the efficacy and safety of these formulations.

## 5 Materials and Methods

### 5.1 Cell Culture

Human urothelial carcinoma cells obtained from the American Type Culture Collection (ATCC) were used for cell culture. The cell lines used were J82 and UM-U-C3, which are high-grade bladder tumor lines. The lines were cultured in Dulbecco’s modified Eagle’s medium (DMEM) (Sigma-Aldrich, Saint Louis, EUA) medium supplemented with 10% fetal bovine serum (Cultilab Ltd.a., Campinas, Brasil), antibiotics (penicillin and streptomycin), and anfotericin at 37 °C in a humidified atmosphere with 5% CO_2_. Control groups of cells that were not exposed to naringenin were included in all assays, and all experiments were performed in triplicate.

### 5.2 Naringenin

Naringenin N5893 (purity >95%) was supplied by Sigma-Aldrich (St. Louis, EUA) and dissolved in DMSO before use, giving a final DMSO concentration of 2% in the well. The concentration of naringenin in DMSO was 10 mg/mL.

### 5.3 Cytotoxicity

The cells were trypsinized and seeded in 24-well plates at a density of 1.0 × 10^5^ cells/mL. They were then exposed to naringenin at concentrations of 12.5, 25, 50, 100, 150, and 200 μM for 24 and 48 h. After treatment, cell viability was evaluated by XTT and sulforhodamine B assay (SRB).^18,41^ The percentage of viable cells was calculated over untreated cells. The time of 48 h was defined as the treatment time for the subsequent experiments due to the greater action of naringenin in almost all of the concentrations used for both cells, information which will be presented in the Section 2.

### 5.4 Clonogenic Survival

Cells were trypsinized and seeded in 24-well plates at 1.0 × 10^5^ cells/mL. Subsequently, the cells were exposed to naringenin (100, 150, and 200 μM) for 48 h. After treatment, the cells were washed with Hanks’ solution and trypsinized, and approximately 1000 cells were plated in 12-well plates, where they remained for 10 days in a CO_2_ oven. The culture medium was then removed, the cells washed again with Hanks’ solution, fixed with 4% formaldehyde for 20 min, hydrated in 100% methanol for 20 min, and stained with a 0.5% crystal violet solution dissolved in 25% methanol. A 33% acetic acid solution was used to remove the dye. The absorbance was measured in a spectrophotometer at 570 nm, and the results were expressed as a percentage of colonies in relation to the control (untreated cells).^42^

### 5.5 Cell Migration and Morphology

The cells were placed in 24-well plates at a cell density of 1.0 × 10^5^ per well and were incubated for 24 h for adherence. After 24 h, the cell monolayer was scraped with a sterile 200 μL tip to create a wound, which was then photographed using an inverted microscope with an attached camera. The cells were then washed with Hanks’ solution and exposed to naringenin (100, 150, and 200 μM) and incubated for 48 h. Once the treatment time had elapsed, the cells were photographed again, and cell migration was observed. The percentage of cell migration was calculated using ImageJ and GraphPad Prism software.^43^

For morphology experiments, the cells were plated on coverslips and incubated for 48 h. After the treatment period, the supernatant was discarded, and the coverslips were stained with rapid panoptic. The coverslips were then dried and affixed to frosted tip slides previously identified by using Entellan (Merck, Darmstadt, Germany). The slides were analyzed under a microscope (Leica-DM 25.000, Wetzlar, Germany) at 100× magnification, using IR 1.515 immersion oil.^44^

### 5.6 Cell Cycle

Cells were plated in 12-well plates at a density of 1.0 × 10^5^ cells and incubated for 24 h to allow adherence. They were then treated with naringenin at concentrations of 100, 150, and 200 μM and incubated for an additional 48 h. After treatment, the cells were washed, trypsinized, and collected with the supernatant into Falcon tubes, followed by centrifugation at 1000 rpm for 10 min. The supernatant was discarded, and the cells were resuspended in 200 μL of propidium iodide labeling solution and then transferred to cytometry tubes. The tubes were incubated on ice for 30 min in the dark. Readings were conducted using a BD FACScalibur (BD Biosciences, Franklin Lakes, NJ) flow cytometer, and the data were analyzed with FlowJo software.^45^

### 5.7 LncRNA Expression Using Reverse Transcription Quantitative Polymerase Chain Reaction (RTq-PCR)

The cells were placed in 24-well plates at a cell density of 1.0 × 10^5^ per well and incubated for 24 h for adherence. After 24 h, the cells were exposed to naringenin (200 μM) and incubated for 48 h. Once the treatment time had elapsed, the cells were trypsinized and centrifuged for 15 min at 1000 rpm. The supernatant was discarded, and the RNA was extracted using the Quick-RNA MicroPrep ZymoSpin IC Columns kit (Zymo Research Califórnia) and quantified using a NanoDrop spectrophotometer (Thermo Scientific, Waltham, MA). The cDNA for the lncRNAs *RP11-36E7.4* and *SBF2-AS1* were produced using the High-Capacity kit (Applied Biosystems, Waltham, MA). Expression was assessed by reverse transcription quantitative polymerase chain reaction (RT-qPCR) using SYBR Green (Thermo Fisher Scientific, Waltham, MA), which was performed with thermocycling conditions for all targets, including a temperature profile at 95 °C for 10 min for initial denaturation, followed by 40 cycles at 95 °C for 15 s and 60 °C for 60 min. The genes *GAPDH* and *HSPCB* were used as reference. The primer sequence as well as the conditions for performing RT-qPCR were made according to Silva et al.^10^

### 5.8 Obtainment of the Nanoemulsions

The nanoemulsion was developed using the phase inversion emulsification method.^28,39^ Naringenin was dissolved in DMSO, after which it was incorporated into the oil phase and subsequently heated at 80 °C. The aqueous phase (80 °C) was introduced into the oil phase, where it was thoroughly agitated at a constant rate of 700 rpm utilizing a mechanical stirrer. The mixture was subjected to constant agitation until it reached a temperature consistent with the surrounding environment. Two formulations were developed: one with chitosan (ChNE NAR) and one without chitosan (NE NAR). In the NE NAR, the aqueous phase consisted exclusively of water, while in the ChNE NAR, the aqueous phase was a solution of deacetylated shrimp shell-derived chitosan (minimum 75% deacetylation) (Sigma-Aldrich, St. Louis, MO). The final composition of the nanoemulsions is listed below: Sorbitan stearate (Span 60—Croda, Campinas, Brazil) (4% w/v), PEG-40 hydrogenated castor oil (Croduret 40—Croda, Campinas, Brazil) (6% w/v), sunflower oil (10% w/v), DMSO (Sigma-Aldrich, St. Louis, MO) (10% w/v), and naringenin (10% w/v). ChNE also contained chitosan 0.5% w/v.

### 5.9 Physical–Chemical Characterization of the Nanoemulsions

The particle size and polydispersity index (PDI) were measured by photon correlation spectroscopy and ζ potential by electrophoretic mobility through Zetasizer (Malvern, model Zetasizer Nano series-Nano ZS). The measurements of particle mean size, PDI, ζ-potential, and pH were conducted for all samples immediately after processing and again at intervals of 1, 7, 14, and 28 days to study the stability of samples maintained at room temperature.^47^

Encapsulation efficiency (EE) was determined using ultracentrifugation/ultrafiltration in microtubes assay, and the drug release study was performed using dialysis membrane with ethanol 50% v/v in phosphate-buffered saline (PBS), pH 6.2, as receptor medium (nano lapachol). The in vitro drug release study was performed by using dialysis membrane (SnakeSkinTM Dialysis Tubing). The nanoemulsions were added to the donor compartment, and it was placed in contact with the receptor medium under stirring at 37 °C. Aliquots were removed from receptor medium at specific intervals (2, 4, 6, 8, 24, and 48 h), and the same volume of a freshly solutions was replaced to maintain the sink conditions.

In EE and release assays, the naringenin was quantified by high-performance liquid chromatography-photodiode array detector (HPLC-DAD). The analysis was performed on a Waters Alliance HPLC-DAD system (Waters, Milford, MA) equipped with a C-18 column (Luna, 4.6 mm × 250 mm, 5 μm particle size) (Phenomenex, Torrance, CA) at 30 °C. The mobile phase consisted of methanol–water acidified with 0.01 M phosphoric acid in a 65:35 (v/v) ratio with isocratic elution at a flow rate of 0.6 mL/min. The injection volume was 10 μL, and the column oven was maintained at 40 °C. Detection was performed at 290 nm, representing the maximum absorption wavelength in the UV spectrum for naringenin.^48^ This method was semi-validated by analyzing the parameters of linearity, specificity, accuracy, and precision according to the International Conference on Harmonization.^49^

### 5.10 Evaluation of Cytotoxicity and Mucoadhesion of the Nanoemulsions

The cytotoxic potential of the nanoemulsions was evaluated for the J82 and U-MU-C3 cell lines according to the previously established methodology for naringenin. The assayed concentrations ranged from 0.003 to 0.050% v/v of the formulation, equivalent to naringenin concentrations of 12.5, 25, 50, 100, 150, and 200 μM for 48 h.

The *in vitro* mucoadhesion was evaluated by preparing a solution with a 1:1 ratio of mucin and the nanoemulsion systems. A volume of 1 mL of mucin, with a concentration of 0.6 mg/mL, was then combined with 1 mL of each nanoemulsion. Subsequently, the mixture was subjected to incubation at 37 °C for 3 h at a speed of 300 rpm.^50^ Subsequent to this interval, the ζ potential was gauged by means of electrophoretic mobility through Zetasizer (Malvern, model Zetasizer Nano series—Nano ZS).

The *ex vivo* mucoadhesion and toxicity were analyzed using freshly extracted porcine urinary bladders obtained from a slaughterhouse. Sections of tissue of 1 × 1 cm^2^ with their mucosal surface facing upward were mounted on glass slides and washed with PBS buffer. Aliquots of 10 μL of each formulation or deionized water (control) were applied onto the bladder mucosa. Aiming evaluate the mucoadhesion, artificial urine has been prepared: Na_2_HPO_4_ (0.055 g/L), NaH_2_PO_4_·H_2_O (0.5 g/L), Na_2_SO_4_ (0.13 g/L), NaHCO_3_ (0.17 g/L), MgSO_4_·7H_2_O (0.5 g/L), CaCl_2_ (0.335 g/L), NH_4_Cl (0.805 g/L), KCl (2.25 g/L), NaCl (3.17 g/L), and urea (12.135 g), pH 6.2. After application of the nanoemulsions, the bladder mucosa was washed three times with 10 mL of artificial urine at a rate of 2 mL/min. After each washing cycle, fluorescence microscopy images were taken again, and ImageJ software was used to analyze the average fluorescence values.^51^

For the histopathological analysis, the tissues with formulations were incubated for 4 h at 37 °C in a humidity chamber. Then, the bladder mucosa was washed to remove excess formulations and fixed in 4% neutral-buffered formalin. Tissue sections of 5 μm thickness were prepared following standard histological procedures, stained with hematoxylin–eosin (HE), and examined using a light microscope. For each formulation, three different bladder tissue samples were analyzed to assess potential tissue damage in healthy mucosa.^46,51^

### 5.11 Statistical Analysis

All of the experiments were carried out in triplicate, and the data obtained were analyzed using GraphPad Prism 8.0. The normal distribution of the data presented was verified using the Shapiro–Wilk test. The results are shown as mean and standard error and were subjected to an analysis of variance (ANOVA), followed by comparison with Dunnett’s post hoc test. A *p*-value of less than 0.05 was considered to indicate a statistically significant difference.
